# Virulence in Murine Model Shows the Existence of Two Distinct Populations of Brazilian *Vaccinia virus* Strains

**DOI:** 10.1371/journal.pone.0003043

**Published:** 2008-08-26

**Authors:** Jaqueline Maria Siqueira Ferreira, Betânia Paiva Drumond, Maria Isabel Maldonado Coelho Guedes, Marcelo Antônio Pascoal-Xavier, Camila Megale Almeida-Leite, Rosa Maria Esteves Arantes, Bruno Eduardo Fernandes Mota, Jônatas Santos Abrahão, Pedro Augusto Alves, Fernando Meireles Oliveira, Paulo César Peregrino Ferreira, Cláudio Antônio Bonjardim, Zélia Inês Portela Lobato, Erna Geessien Kroon

**Affiliations:** 1 Laboratório de Vírus, Departamento de Microbiologia, Instituto de Ciências Biológicas, Universidade Federal de Minas Gerais.Avenida Antônio Carlos, Belo Horizonte, Brazil; 2 Laboratório de Vírus, Departamento de Medicina Veterinária Preventiva, Escola de Veterinária, Universidade Federal de Minas Gerais, Belo Horizonte, Brazil; 3 Laboratório de Neuro-Imuno Patologia Experimental, Departamento de Patologia Geral, Instituto de Ciências Biológicas, Universidade Federal de Minas Gerais, Belo Horizonte, Brazil; AIDS Research Center, Chinese Academy of Medical Sciences and Peking Union Medical College, China

## Abstract

Brazilian *Vaccinia virus* had been isolated from sentinel mice, rodents and recently from humans, cows and calves during outbreaks on dairy farms in several rural areas in Brazil, leading to high economic and social impact. Some phylogenetic studies have demonstrated the existence of two different populations of Brazilian *Vaccinia virus* strains circulating in nature, but little is known about their biological characteristics. Therefore, our goal was to study the virulence pattern of seven Brazilian *Vaccinia virus* strains. Infected BALB/c mice were monitored for morbidity, mortality and viral replication in organs as trachea, lungs, heart, kidneys, liver, brain and spleen. Based on the virulence potential, the Brazilian *Vaccinia virus* strains were grouped into two groups. One group contained GP1V, VBH, SAV and BAV which caused disease and death in infected mice and the second one included ARAV, GP2V and PSTV which did not cause any clinical signals or death in infected BALB/c mice. The subdivision of Brazilian *Vaccinia virus* strains into two groups is in agreement with previous genetic studies. Those data reinforce the existence of different populations circulating in Brazil regarding the genetic and virulence characteristics.

## Introduction

*Vaccinia virus* (VACV) is the prototype virus of the family *Poxviridae* and it was used as a live vaccine during the smallpox vaccination campaign. The World Health Organization declared smallpox, the contagious and deadly disease caused by *Variola virus*, eradicated in 1980 [1]. In spite of that, the poxviruses still have a great impact in human and animal health due to the existence of emerging and reemerging zoonotic agents in this family. Cases of *Cowpox virus* human infections have been reported in Europe [2], [3]. *Monkeypox virus* is endemic in the tropical rainforest villages of Central and Western Africa, causing sporadic cases in humans. Moreover, 37 confirmed human cases have been recently reported in the United States [4], [5].Outbreaks of *Buffalopox virus* affecting buffaloes, cows and humans have been reported in countries as India, Egypt and Bangladesh [6], [7].

In Brazil, several strains of VACV have been isolated since the 60's. Some of these strains were isolated from sentinel mice, such as *Cotia* [8], [9] and SPAn232 (SAV) [10]. BeAn58058 (BAV) was isolated from a rodent of *Oryzomis* genus [11] while Belo Horizonte (VBH) was isolated from an outbreak affecting laboratory mice [12]. Since the 90's, bovine vaccinia outbreaks have been reported occurring in many rural regions in Brazil. All the VACV isolates, such as Cantagalo virus, Araçatuba virus, Guarani P1 virus, Guarani P2 virus, Passatempo virus, were equally pathogenic to cattle regarding the macroscopic evolution pattern of lesions. Initial acute lesions were associated with the appearance of erythema evolving to vesicles, papules and pustules, ending with the formation of scabs. Those lesions were mainly observed on the teats of cows and on the nose and mouth of calves [13]–[18]. In all those bovine outbreaks, human infections were also reported. In humans, hands and face were the mainly affected regions and the lesions were similar to those observed in cows and calves. Lymphadenopathy was also frequently observed in humans [14], [16]. The vaccinia outbreaks cause economic loss mainly due to decrease in milk production and to the fact that milkers often stand back from working [13], [16], [19].

Due to the importance of VACV in human and animal medicine, some studies have been conducted in order to characterize those Brazilian vaccinia strains (BR-VACV). It was demonstrated that there is genetic variability among them which could reflect on different biologic characteristics; however there is little information about the virulence patterns of BR-VACV. Leite and collaborators (2007) demonstrated based on the structure of the A-type inclusions *(ati)* gene that there are three different groups of BR-VACV. Phylogenetic analyses based on sequences of genes A56R, B19R, E3L [18] and of ten genes related with virulence and host-range [20] demonstrated the existence of two well defined groups of BR-VACV [18], [20].

The origin of VACV is unknown [2] and the most appropriate animal model for studying VACV virulence is still uncertain. Different animal models have been used to study poxvirus virulence and the murine model is considered a good animal model due to the availability of well-defined inbred strains [21]. Vaccinia virus infection has been studied after different infection routes, such as intravenous, intraperitonial, intradermal, intracranial, and intranasal [22]–[28]. The intranasal route is the natural way of infection by *Variola virus* and it is considered the most frequent route of *Orthopoxvirus* transmission [29]. Moreover, it had already been described that intranasal and intracranial inoculation of moderate doses of VACV strain Western Reserve (WR) can cause systemic infections that permit the observation of differences in virulence patterns among different strains of VACV [23], [27], [30], [31]. Thus, due to the existence of genetic diverse populations of VACV in Brazil and to the lack of studies focused on their biologic characteristics, the aim of the present work was to study the virulence and tropism patterns of different BR-VACV, after intranasal infection of BALB/c mice.

## Materials and Methods

### Viruses and cells

Brazilian *Vaccinia virus* strains used in this study are shown in Table 1. The *Vaccinia virus*, strain Western Reserve (VACV-WR), kindly provided by Dr. C. Jungwirth (Universität Würzburg, Germany), was used as a highly virulent reference strain in murine model by intranasal infection [23], [32]. Lister Butantan strain (LST-BUT), kindly provided by Instituto Butantan (São Paulo, Brazil) is a vaccine strain and it was used as a low virulence reference strain [27], [32]. All virus stocks were grown in Vero cells (ATCC CCL-81) cultivated in Eagle's minimum essential medium (MEM) supplemented with 5% of fetal calf serum (Cultilab, Brazil) and antibiotics, at 37°C in 5% CO_2_ atmosphere. Viruses were purified on sucrose gradients as described elsewhere [33].

**Table 1:** Brazilian *Vaccinia virus* strains used in this study.

Strain, isolate (abbreviation)	Source	Isolation Year	Geografic isolation (State)	Reference
BAV	Rodent, *Oryzomis sp.*	1963	Para	Da Fonseca *et al.*, 1998
SAV	Rodent, sentinel	1965	São Paulo	Da Fonseca *et al.*, 2002
VBH	Rodent, Balb-c mice	1993	Minas Gerais	Trindade *et al.*, 2004
ARAV	*Bos taurus*	2000	São Paulo	Trindade *et al.*, 2003
GP1V	*Bos taurus*	2001	Minas Gerais	Trindade *et al.*, 2006
GP2V	*Bos taurus*	2001	Minas Gerais	Trindade *et al.*, 2006
PSTV	*Bos taurus*	2003	Minas Gerais	Leite *et al.*, 2005

* BAV, BeAn58058; SAV, SpAn232 virus; VBH, *Belo Horizonte virus*; ARAV, *Araçatuba virus*; GP1V Guarani P1 virus; GP2V: Guarani P2 virus; VACV-WR, *Vaccinia virus* Western Reserve.

### Animal experimentation

For all animal experimentation, four-weeks-old male BALB/c mice were used. Mice were housed in filter-top micro isolator cages and provided with commercial mouse feed and water *ad libitum*. All the animal experimentation was carried out in accordance with regulations and guidelines of Committee of Ethics for Animal Experimentation from Universidade Federal de Minas Gerais/Brazil.

BALB/c mice were anesthetized by intraperitonial injection of ketamine and xilazine (3.2 mg and 0.16 mg/mice, in phosphate buffered saline (PBS) 0.9%, respectively) and inoculated by intranasal route. Mice belong to negative control group were inoculated with 10 µl of PBS [32].

### Survival rate and lethal dose (LD_50_) in mice

For the survival rate analysis, mice were infected with 10^6^ PFU (plaque forming units) of BR-VACV BeAn58058 (BAV), SpAn232 virus (SAV), *Belo Horizonte virus* (SAV), *Araçatuba virus* (SAV), *Guarani P1 virus* (GP1V), *Guarani P2 virus* (GP2V), *Passatempo virus* (PSTV) and with the control strains VACV-WR and LST-BUT. To establish the lethal dose of 50% of the animals (LD_50_) mice were infected with virus titers ranging from 10^3^ to 10^8^ PFU of BAV, SAV, VBH, GP1V and control strain in a total volume of 10 µl which was dropped by intranasal route. For both experiments, mice were daily observed during 20 days post inoculation (d.p.i.) or until their death. Mice that survived until day 20 p.i. were euthanized.

### Vaccinia virus tropism in mice

Groups of four, BALB/c mice were infected with 10^6^ PFU of each purified virus strain in a total volume of 10 µl which was dropped by intranasal route. Mice were daily weighted and those ones that had lost more than 25% of their initial body weight were euthanized with an overdose of anesthetics (ketamine+xilazine). Those animals had their organs (trachea, lungs, heart, kidneys, liver, brain and spleen) asseptically removed after euthanasia. Those mice that survived were euthanized on day 12 p.i. and their organs and blood were also collected.

In order to compare the replication of all VACV strains in lungs, (a main site of viral replication in this model), a virus multiplication kinetic was made at days 1, 3 and 5 post intranasal infection with 10^6^ PFU of BAV, SAV, VBH, GP1V, ARAV GP2V, PSTV, and with the control strains VACV-WR and LST-BUT.

### Virus titration

The organs were macerated in MEM (Gibco, USA) and centrifuged at 2000×g for 3 min, 4°C. Supernatant fluids from macerated organs were collected and virus titer (PFU/g) was determined by plaque forming assay in Vero cell culture [34].

### Neutralization test

Blood samples were collected from BALB/c mice just before euthanasia. The serum was heated at 56°C for 30 min and used in virus neutralization assay as described previously [28]. The neutralization titer was expressed as the reciprocal of the highest dilution that exhibited 50% of plaque formation inhibition, when compared to cells infected with VACV WR (positive control).

### Histopathologic analysis

Fragments of lungs were fixed in 10% neutral-buffered formalin and routinely processed for paraffin embedding. Sections (5 µm) were cut on a standard rotary microtome and placed on glass slides for staining with hematoxylin and eosin (H&E). Other unstained sections were used for immunohistochemical analysis to localize viral antigen. Histological sections were examined by an experienced pathologist and classified in mild, moderate or severe according to lesion extent and severity.

### Immunohistochemistry

For immunohistochemistry analysis, deparaffinized lung sections were washed in PBS for 15 min at room temperature. Antigen retrieval was performed by incubating the slides in a specific solution (Target Retrieval Solution, S1700, Dako Corporation, USA) at 98°C for 20 min. Endogenous peroxidase activity was abolished by incubation with 3.5% H_2_O_2_ for 30 min, which was followed by incubation with 1∶20 normal goat serum (NGS) and 2% bovine serum albumin (BSA) for 30 min each at room temperature in humid chamber. Primary rabbit anti-H3L protein from VACV [35] was used at the dilution 1∶10.000, and the antibody incubation was performed overnight at 4°C in humid chamber. Secondary biotinylated antibody was goat anti-rabbit, followed by streptavidin-peroxidase complexes (LSAB2 system – HRP, DAKO, USA). The reaction was visualized by incubating the section with 3,3-diaminobenzidine tetrahydrochloride (Sigma, St. Louis, MO) and counter staining with hematoxylin. Tissues from non-infected animals were used as negative controls. Technical control was performed by the omission of the primary antibody. The sections were examined by Olympus BX51 microscope and digital images were acquired for documentation.

### Polymerase chain reaction (PCR)

For viral DNA detection, a semi-nested PCR for vaccinia growth factor (*vgf*) gene amplification was used (unpublished data). In the first reaction, 2 µl of clarified tissue samples were used as template without previous DNA extraction, added to 18 µl of the PCR reaction mixture. PCR products from the second round of amplification were fractionated by electrophoresis on 8% PAGE and silver stained.

### Statistical analysis

Significance variations were calculated using the Student's t-test (*p*≤0.05: statistical significance). Analysis of significant differences on weight curves and survival were done, respectively, by Wilcoxon paired test (*p*≤0.05: statistical significance) and the log-rank test (*p*≤0.05: statistical significance). Data were analyzed for individual mice. Statistical analysis was performed using GraphPad Prism Software, version 3.0. (GraphPad Software, Inc., San Diego, CA).

## Results

### Clinical signs

**Figure 1:**
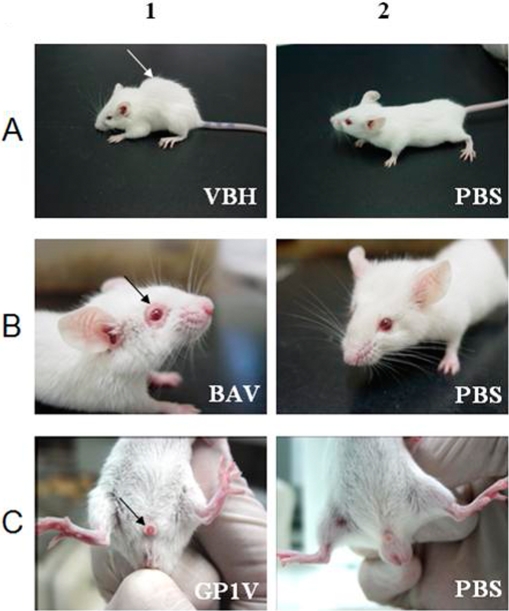
Clinical signs of BALB/c BR-VACV infected mice. Seven BALB/c mice were intranasally infected with 10^6^ PFU/10 µl and observed daily. 1A) Ruffling fur and arching back in mice infected with VBH on day 3 p.i. 1B) Periocular alopecy caused by BAV on day 4 p.i. 1C) Penis with balanopostitis on day 3 p.i. of mice infected with GP1V. 2A, 2B, 2C). No clinical signals were observed in control mice inoculated with PBS.

Seven BALB/c mice were infected with 10^6^ PFU of each VACV strain by intranasal route. The induction of clinical signals such as ruffling fur and arching back was observed in all animals inoculated with VBH (Figure 1A), BAV, SAV, GP1V and the control VACV-WR within days 2–3 p.i. Five mice inoculated with BAV and SAV strains presented periocular alopecy and closed eyes with inflammation on day four p.i. (Figure 1B). Mice inoculated with GP1V presented penis with balanopostitis on day three p.i. (Figure 1C), but those animals recovered from it after a few days.

**Figure 2:**
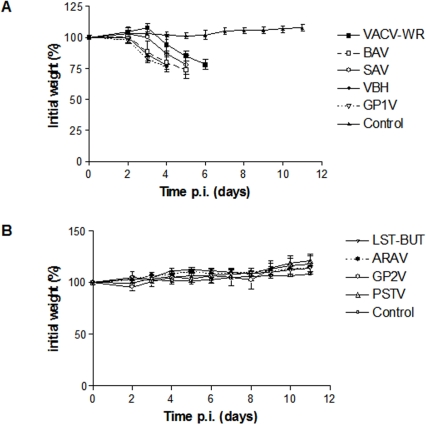
Body weight of BALB/c mice infected with BR-VACV. Groups of mice (n = 4) were inoculated with 10^6^ PFU of VACV by the intranasal route: (A) WR, BAV, SAV, VBH GP1V, and (B) LST-BUT, ARAV, GP2V and PSTV. The weight of the mice was determined daily and those that lost more than 25% of their initial weight were euthanized. The percentages of median weight relative to their initial weight were plotted. The weight of uninfected mice (control) was followed as well (A, B). The error bars indicate the standard deviations. Significance variations were calculated using the Student's t-test (P≤0,05: statistical significance).

The clinical signals of mice infected with BAV, SAV, GP1V, VBH and the control strain WR were associated with severe weight loss up to 25% on day five (WR), four (BAV and SAV) or three p.i. (VBH and GP1V) (Figure 2A). On the other hand, no clinical signals were observed in mice infected with PSTV, ARAV, GP2V or with the other control strain LST-BUT, as well as the control mice, inoculated with PBS. None of those animals died or lost weight (Figure 2B) during the twelve days of observation.

### Survival rate and lethal dose (LD_50_)

**Figure 3:**
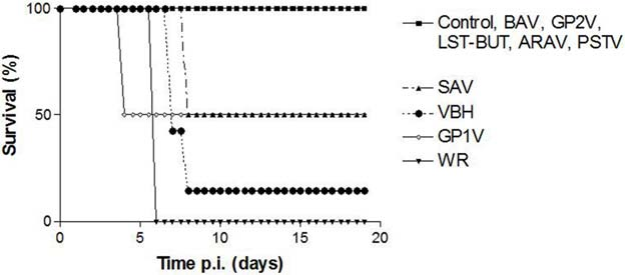
Survival curve of mice infected with BR-VACV. Groups of seven mice were inoculated by the intranasal route with 10^6^ PFU of BR-VACV BAV, GP2V, control LST-BUT, ARAV, PSTV, SAV, VBH, GP1V and control WR. The control group was inoculated with PBS. Mice were monitored daily until day 20 p.i.

To establish the survival rate, seven BALB/c mice were infected with 10^6^ PFU of each VACV strain by intranasal route and observed for 20 days. All mice infected with WR died on sixth day p.i., 50% of mice infected with GP1V died on fourth day p.i. and 80% of mice infected with VBH and SAV died on days eighth and ninth p.i., respectively (Figure 3).

Since strains BAV, SAV, GP1V, VBH and WR were the only ones able to induce clinical signals and/or death of mice infected with 10^6^ PFU, we performed an assay to estimate the lethal dose of those VACV strains. For that, mice were infected by intranasal route with virus doses ranging from 10^3^ to 10^8^ PFU of BAV, SAV, GP1V, VBH and WR. According to the LD_50_ estimation, VBH was the most virulent strain (LD_50_ = 10^3^ PFU), followed by WR (LD_50_ = 10^4^ PFU), SAV and GP1V (LD_50_ = 10^6^) and BAV (LD_50_ = 5×10^7^ PFU) (Table 2).

**Table 2:** Lethal dose of 50% of mice infected by intranasal route.

VACV strains	LD_50_ (PFU)*
VBH	10^3^
WR	10^4^
GP1V	10^6^
SAV	10^6^
BAV	5×10^7^

* Groups of mice (n = 7) were followed for 20 days pi or until their death with doses ranging from 10^3^ until 10^8^ PFU/ml.

### Neutralizing antibodies detection

Neutralizing antibodies with titers ranging from 1∶40 to 1∶60 were detected in mice infected with LST-BUT, ARAV, GP2V and PSTV. Since mice infected with 10^6^ PFU of WR, BAV, SAV, GP1V and VBH strains died or were euthanized shortly after infection because they had lost more than 25% of initial weight body it was not possible to perform neutralization assays with clinical samples from those mice. Moreover, neutralizing antibodies could not be detected in sera in initial days post infection of mice infected with WR, BAV, SAV, GP1V and VBH. Neutralizing antibodies were also not detected in mice inoculated with PBS.

### VACV replication in different organs

**Figure 4:**
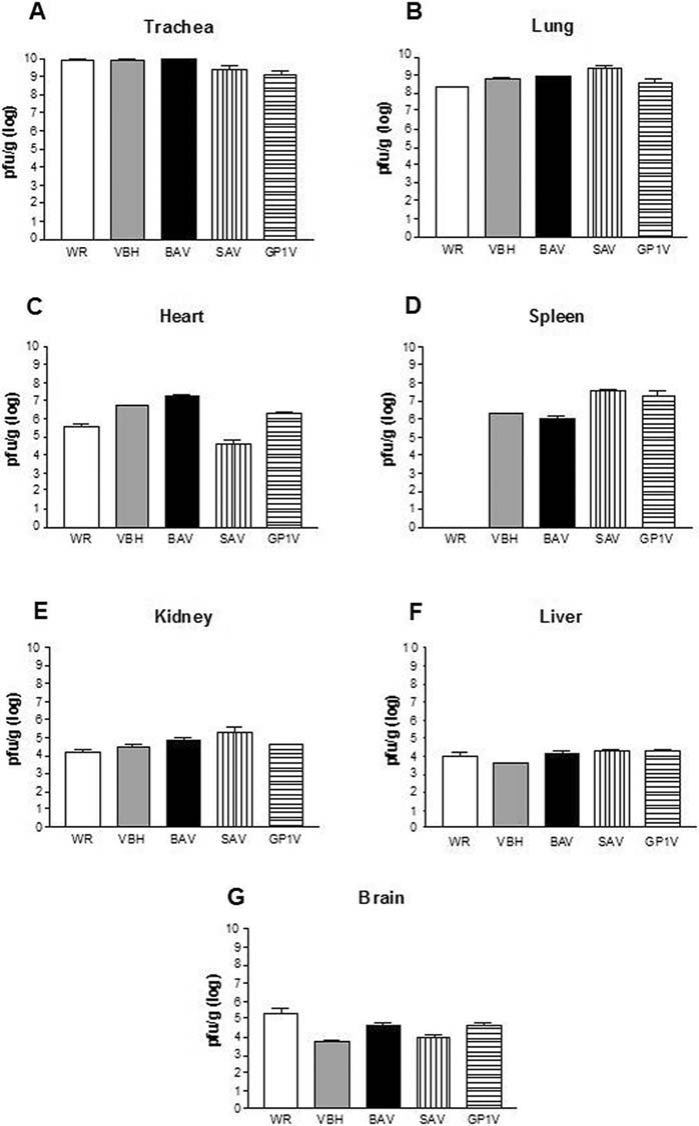
Viral titers in organs of mice infected with BR-VACV. Mice were infected by the intranasal route with 10^6^ PFU/10 µl with BR-VACV strains: VBH, BAV, SAV, GP1V and VACV-WR. Organs were collected and titrated on the day that 25% weight loss occurred and the mice were euthanized. The results presented were obtained by the average titers from three mice. Titers were calculated as PFU/g tissue.

Infectious virus particles were detected in trachea, liver, spleen, lungs, heart, brain and kidneys from mice infected with BAV, SAV, GP1V, VBH and the control VACV-WR on the day that animals presented 25% of weight loss (Figure 4). The highest titers of virus were observed in trachea, ranging from 10^8^ PFU/g of organ (mice infected with VBH) to 10^9^ PFU/g (mice infected with WR, SAV, GP1V and BAV). In the lungs, viral titers ranged from 10^8^ PFU/g (mice infected with WR, BAV, GP1V and VBH) to 10^9^ PFU/g (mice infected with SAV) The only organ in which no virions were detected by the method of plaque forming units was the spleen of mice infected with VACV WR, but virus was detected by PCR in that organ. In all organs of mice infected with ARAV, GP2V, PSTV and control LST-BUT no viral infectious particles were detected.

### Virus replication kinetic in the lungs

**Figure 5:**
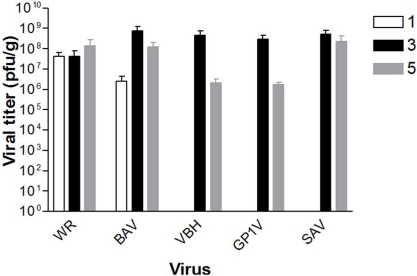
Kinetics of BR-VACV replication in the lungs of infected mice. Mice were inoculated by the intranasal route with 10^6^ PFU/10 µl of VBH, BAV, SAV, GP1V and VACV-WR. Viral titers were estimated in lungs on days 1, 3 and 5 p.i. Titers are expressed as PFU/g tissue. The results presented were obtained by the average titer in organs from three mice. The error bars indicate the standard deviations.

The replication kinetics of VACV strains in the lungs was done on days 1, 3 and 5 post intranasal inoculations of 10^6^ PFU of BAV, SAV, GP1V, VBH, ARAV, GP2V, PSTV and control strains WR and LST-BUT. In mice infected with WR and BAV, infectious particles were detected the lungs from day 1 to day 5 p.i. Infectious particles of VBH, GPV1V and SAV were detected in the lungs from the day 3 until day 5 p.i. (Figure 5). No infectious viral particles were detected in any of the analyzed days in the lungs of mice infected with ARAV, GP2V, PSTV or LST-BUT.

### Histopathological and Immunohistochemistry

**Figure 6:**
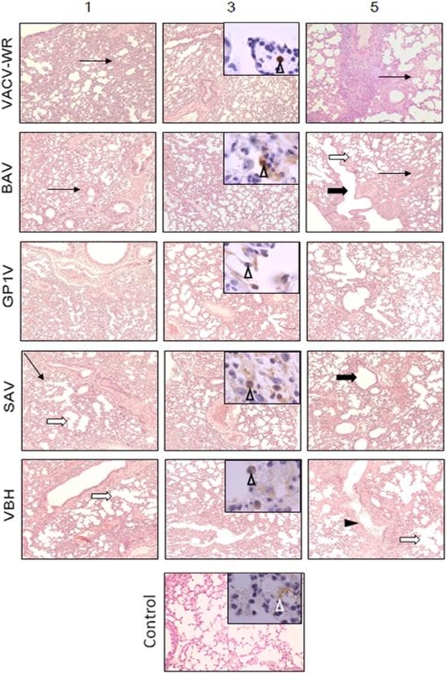
Histological and immunohistochemical analysis of lungs from WR, BAV, GP1V, SAV and VBH VACV infected mice. Histological sections of lungs from mice infected with 10^6^ PFU of VACV were performed on days 1, 3 and 5 p.i. and positive anti-H3L expression was evident in inflammatory cell cytoplasms (inserts, arrow-heads) on day 3 p.i in mice infected with WR, BAV, GP1V, SAV and VBH. Intensity of lesions varied from severe to moderate during the five-day study period. Interstitial pneumonitis was observed in mice infected with all virus strains. Thickening of the interalveolar septa (thin arrows) was observed mainly in mice infected with VACV-WR and BAV, and characterized by mild or moderate mononuclear inflammatory cell infiltrate. Alveolar enlargement (white arrows) due to diffuse alveolar wall rupture and cystic structures lined by bronchial-type epithelium (black arrows) were observed in lungs of mice infected with GP1V, SAV and VBH. Extensive necrosis of bronchial mucosa (black arrow-head) in mice infected with VBH was observed at the fifth day p.i. No lesions or specific immunostaining (white arrow-heads) were detected in the lungs of non-infected mice. Original magnifications: ×35 and ×350 (inserts).

**Figure 7:**
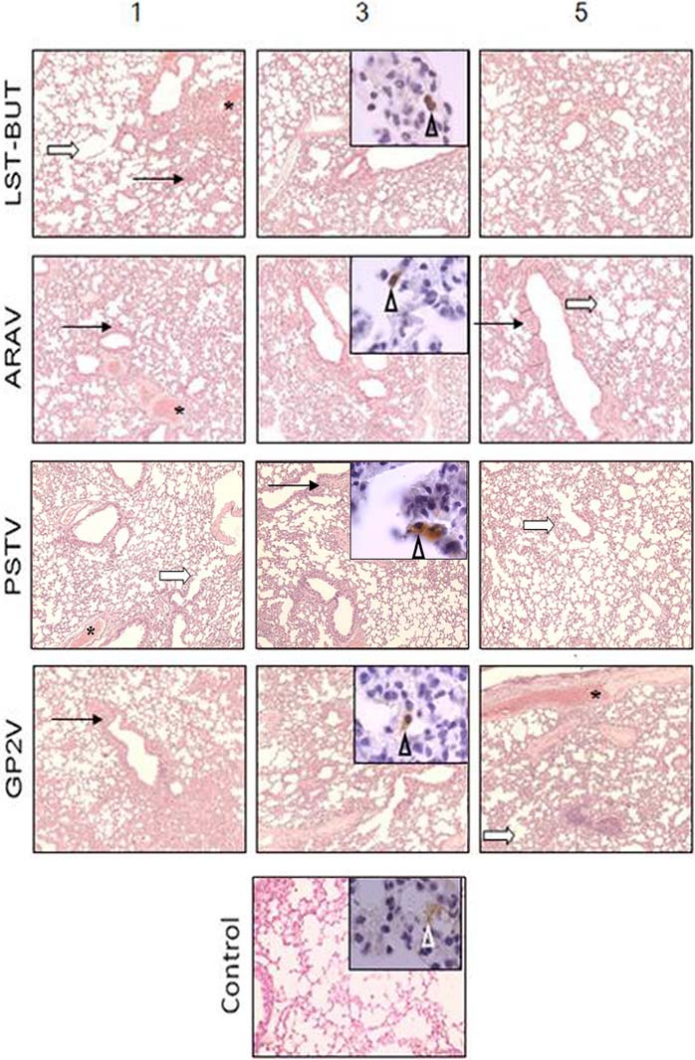
Histological and immunohistochemical analysis of lungs from LST-BUT, ARAV, PSTV and GP2V VACV strains infected mice. Histological sections of lungs from mice infected with 10^6^ PFU of VACV were performed on days 1, 3 and 5 p.i. and positive anti-H3L expression was evident in inflammatory cell cytoplasms (inserts, arrow-heads) on day 3 p.i in mice infected with LST-BUT, ARAV, PSTV and GP2V. Focal interstitial pneumonitis ranging from moderate to mild was observed in mice infected with all viruses. Alveolar enlargement (white arrows) due to diffuse alveolar wall rupture and focal interalveolar septa thickening (long arrows), were observed in lungs of mice infected with LST-BUT, ARAV, PSTV and GP2V. Vascular congestion, and focal hemorrhage (*) were frequently detected. No lesions or immunostaining were detected in non-infected mice lungs (see Control in Figure 6). Original magnifications: ×35 and ×350 (inserts).

The histopathological analyses of lungs were performed and mice infected with all VACV strains had some degree of pneumonia. Severe to moderate lesions were observed in the lungs of mice infected with BAV, GP1V, SAV, VBH and the control VACV-WR (Figure 6). In mice infected with ARAV, PSTV, GP2V and the control strain LST-BUT, mild to moderate lesions were observed in the lungs (Figure 7). All mice developed interstitial pneumonia and bronchiolitis. Lesions in lungs were degenerative, necrotizing, hemorrhagic, and contained predominantly lymphocytic and histiocytic interstitial infiltrates. Hyperplasia of bronchial epithelial cells, fibroblasts, and pneumocytes were also observed. Although all animals had lung lesions, there were differences regarding the distribution and progression patterns of lesions. Pulmonary lesions were diffuse, severe to moderate on days 1, 3 and 5 p.i. in mice infected with BAV, GP1V, VBH, SAV and control strain WR (Figure 6). Interalveolar septa thickening and alveolar collapse due to mild or moderate mononuclear inflammatory cell infiltrate were observed mainly in mice infected with WR and BAV. Alveolar wall rupture and bronchioles destruction with cystic spaces formation were observed in mice infected with GP1V, SAV and VBH. Mucosal epithelial necrosis was also observed in mice infected with VBH and WR. On the other hand, pulmonary lesions were focal and mostly mild in mice infected with ARAV, GP2V, PSTV and the control strain LST-BUT (Figure 7). Hemorrhagic areas were frequently noticed. No lesions were observed in lungs of mice inoculated with PBS.

Immunohistochemistry showed anti-H3L positivity in lungs. Positive H3L expression was evident in cytoplasm of inflammatory cells of lungs from mice infected with all strains (Figure 6, 7). Strong immunopositivity against H3L was present in areas of intense inflammatory infiltrate.

## Discussion

Brazilian VACV strains showed different virulence profiles after intranasal infection of BALB/c mice. Based on their biologic characteristics the studied VACV strains could be divided into two main groups corroborating the genetic data from previous studies [20], [36]. The first one grouped the high virulent strains SAV, VBH, BAV, GP1V and the control VACV-WR. The second group was represented by the low virulent strains ARAV, GP2V, PSTV and the vaccine strain LST-BUT. Previous studies had demonstrated that VACV WR is a high virulent strain and since LST-BUT is a vaccine strain^1^, it was expected that it present a low virulent pattern [27].

The VACV strains SAV, VBH, GP1V, BAV and VACV-WR caused severe morbidity, with clinical signals such as ruffled fur, arching back and weight loss in infected mice (Figure 2). Those strains showed the highest viral titers in the initial sites of infection as trachea and lungs, but also presented a broad tropism in BALB/c mice, spreading to other organs, including brain, liver, kidneys, heart and spleen. Similar results were observed by Abdalrhman and collaborators (2006), when mice infected with 5×10^5^ PFU of VACV-WR by intranasal route had a weight loss at the third day p.i. reaching 25% of weight lost at the fifth day p.i. The highest viral titers were observed in the lungs, but virions were also detected in liver, kidneys, heart and brain. The VBH strain presented the highest LD_50_ (10^3^ PFU) and was the strain that induced 25% of body weight loss sooner than other strains, showing the highest virulence pattern among all the studied strains (Table 2).

The observation of clinical signs in addition to the presence of virions in different organs gives support to the idea that SAV, VBH, GP1V, BAV and control VACV-WR strains are able to cause systemic disease after intranasal infection. Lung tissues affected by pneumonia were stained by anti-H3L antibody confirming the presence of SAV, VBH, GP1V, BAV and the control VACV-WR in the lungs of mice after intranasal infection. These findings are in accordance with studies showing pneumonia, characterized by severe alveolar edema, acute necrotizing bronchiolitis and peribronchiolitis as well as neutrophilic infiltrate in the intersticium of lungs from mice infected with VACV WR [29], [37]. Differences regarding the mortality were observed in this group; except for BAV, all other strains caused death of infected animals after infection with 10^6^ PFU by intranasal route. It is interesting to note that BAV was isolated from a naturally infected rodent and one could propose that its morbidity pattern could be a consequence of the adaptation of BAV to rodents [11], but more studies involving these strains are necessary to elucidate that.

The second group was represented by ARAV, GP2V, PSTV and the control strain LST-BUT that did not cause clinical signs, weight loss or death of infected mice. Previous studies already showed that infection of in BALB/c mice inoculated with 5×10^5^ PFU of Lister (Elstree) also did not induce weight loss in those animals. In contrast as in this work where infectious particles were detected in lungs of mice inoculated with LST-BUT or ARAV, GP2V and PSTV those studies [23], [27], [32] demonstrated the presence of infectious viral particles in the lungs of mice infected with Lister (Elstree) by plaque assay in Vero cells, as we did. Although the strain LST-BUT is derived from Lister (Elstree), there is no apparent documentation of plaque purification before the distribution of the Lister strain around the world [38], and it could be that different virus populations were favored or evolved in response to different growing conditions in many laboratories. In fact genetic differences have already been demonstrated among Lister and Lister derived strains [38], including LST-BUT [20]. On the other hand, infectious viral particles of Lister Elstree could not be detected in liver, kidneys, heart and brain of infected mice [23], [27], [32], as was observed in this work, since no infectious particles were detected in organs as trachea, lungs, brain, liver, kidneys, heart and spleen by plaque assay in Vero cells when mice were inoculated with LST-BUT or ARAV, GP2V and PSTV.

Despite focal and mild lesions observed in lungs of mice infected with ARAV, GP2V, PSTV and the control strain LST-BUT, no infectious particles were detected in those organs by plaque assay in Vero cells. As mentioned, low replication rates of those strains and the little sensitivity of plaque assay could explain this fact, since histological alterations and immunopositivity against the virus confirmed its presence in the lungs. The absence of clinical signals, the presence of lesions in lungs associated with the presence of virus in that organ and the detection of neutralizing antibodies point to the development of a sub-clinical infection in mice after inoculation of GP2V, ARAV, PSTV and the control strain LST-BUT by intranasal route.

This grouping of Brazilian VACV strains based on biological characteristics is in agreement with previous phylogenetic studies based on the analysis of genes related with tropism and virulence. Some studies showed that strains BAV, SAV, VBH and GP1V are closely related to each other and to VACV WR, on the other hand, strains PSTV, ARAV and GP2V are closely related to each other. [20], [36] Drumond and collaborators (2008) showed that BAV, SAV, VBH, GP1V, ARAV, GP2V and PSTV are not close to from LST-BUT, one vaccine strain used during Smallpox Eradication Campaign, in Brazil. In this work we showed that in addition to the genetic differences, BAV, SAV, VBH and GP1V are also biologically different from LST-BUT, in Balb/c mice after intranasal infection. On the other hand, ARAV, GP2V and PSTV presented similar biological characteristics to LST-BUT regarding morbidity, mortality, virus replication, lesions development in the lungs and also the neutralizing antibodies levels.

Previous studies had demonstrated that although GP1V and GP2V were isolated from cows in two neighbor rural properties during the same bovine vaccinia outbreak, they were genetically divergent [7], [18], [20], [39] Our study confirmed that the divergence between GP1V and GP2V also occurs at the biological phenotypic level. While GP2V did not cause weight loss or death in BALB/c, GP1V caused weight loss that culminated in the death of infected mice.

Although strains from one group, ARAV, GP2V and PSTV were all isolated from cows during bovine vaccinia outbreaks, strains belonging to the other group SAV, BAV and VBH were isolated from rodents while GP1V was isolated from a cow. Thus, analyzing these results, there is no obligatory correlation between the host species where the virus was isolated and the biological characteristics of VACV strains studied here, which is in agreement with previous phylogenetic studies [36], [39].

VACV has already been isolated from rodents in nature, as exemplified by BAV, and those animals could be important to the maintenance and transmission of VACV in nature, as observed for other *Orthopoxvirus* [40]–[42]. Other studies of our group suggest that excretes from infected mice could be a source of virus elimination and transmission (unpublished data). This work demonstrated that BR-VACV strains can cause different types of infections in mice: clinical (SAV, BAV, GP1V and VBH) and sub-clinical (PSTV, ARAV and GP2V). Rodents with clinical and sub-clinical disease could play an important role in VACV maintenance and transmission to different hosts in nature, what should be further investigated.

Our results confirmed that there are different populations of Brazilian VACV strains regarding their virulence, circulating in nature even during the same bovine vaccinia outbreak, such as GP1V and GP2V. Regardless the fact that ARAV, GP2V and PSTV exhibit a low virulence pattern causing sub-clinical disease in BALB/c mice, they were associated with bovine vaccinia outbreaks in which in cows, calves and humans were infected, leading to economic and health problems. Moreover, the co-circulation of VACV strains with different biological characteristics, could favor events such as recombination that could give origin to more virulent strains. In conclusion, this work brings new and relevant data about the biology of VACV strains and reinforce the need of more studies to elucidate the genotypic and phenotypic characteristics of those Brazilian VACV strains and not only in BALB/c model, but also in other rodents and bovines.
